# Microbial Ecology of Granular Biofilm Technologies for Wastewater Treatment: A Review

**DOI:** 10.3390/microorganisms12030433

**Published:** 2024-02-20

**Authors:** Aurora Rosa-Masegosa, Alejandro Rodriguez-Sanchez, Susanna Gorrasi, Massimiliano Fenice, Alejandro Gonzalez-Martinez, Jesus Gonzalez-Lopez, Barbara Muñoz-Palazon

**Affiliations:** 1 Department of Microbiology, Faculty of Pharmacy, University of Granada, 18071 Granada, Spain; aurorarm@ugr.es (A.R.-M.); arod@ugr.es (A.R.-S.); agon@ugr.es (A.G.-M.); jgl@ugr.es (J.G.-L.); 2 Department of Ecological and Biological Sciences (DEB), University of Tuscia, 01100 Viterbo, Italy; gorrasi@unitus.it (S.G.); fenice@unitus.it (M.F.)

**Keywords:** microbiota, aerobic granular sludge, anaerobic granular sludge, anammox, microbial ecology, nutrient removal, organic carbon degradation

## Abstract

Nowadays, the discharge of wastewater is a global concern due to the damage caused to human and environmental health. Wastewater treatment has progressed to provide environmentally and economically sustainable technologies. The biological treatment of wastewater is one of the fundamental bases of this field, and the employment of new technologies based on granular biofilm systems is demonstrating success in tackling the environmental issues derived from the discharge of wastewater. The granular-conforming microorganisms must be evaluated as functional entities because their activities and functions for removing pollutants are interconnected with the surrounding microbiota. The deep knowledge of microbial communities allows for the improvement in system operation, as the proliferation of microorganisms in charge of metabolic roles could be modified by adjustments to operational conditions. This is why engineering must consider the intrinsic microbiological aspects of biological wastewater treatment systems to obtain the most effective performance. This review provides an extensive view of the microbial ecology of biological wastewater treatment technologies based on granular biofilms for mitigating water pollution.

## 1. Introduction

Microorganisms are ubiquitous in every habitat on Earth, and they are the main drivers of the exchange of carbon, oxygen, nitrogen, phosphorous, and sulfur in the biogeochemical cycles that comprise the basis of life. The mixed microbial populations interact, connect, influence, and alter the biochemical and physiological processes occurring on the planet [1]. The microbial ecology of bioengineering systems is a key factor in the performance of bioprocesses. The design and operation of biotechnological approaches must consider their intrinsic microbiological aspects [2]. Furthermore, the understanding of the microbial communities and their roles are basic for the profitable management of biological resources [3]; thus, microbial ecosystems are considered functional entities in processes based on the metabolic pathways of mixed-culture microorganisms such as wastewater treatment (WWT) systems, which are among the most widespread uses of microbial communities currently [1]. The processes in WWT are not carried out by specific genera but by complex microbial communities that are usually characterized by parameters such as biomass concentration, the number of cells, sludge age, or kinetics, all of which are studied from the engineering point of view [4].

Furthermore, the microbiological aspects tend to be focused on the cultivation of isolated microorganisms that play a role in the destabilization of biomass, such as filamentous bacteria [5]. It is important to note that new molecular biology tools have allowed for the identification of the microbiome of mixed cultures, having a major impact on the field of microbial ecology. Next-generation sequencing that employs metabarcoding or shot-gun analysis has allowed researchers to delve deeper into the relationships between the microorganisms of a community in bioprocesses, offering capabilities for analyzing DNA and RNA molecules in a high-throughput and cost-effective manner [6]. Nowadays, molecular biology facilitates differentiating the communities for each technology and operational condition, demonstrating which microorganisms can proliferate and which ones are not competitive for survival.

**Figure 1:**
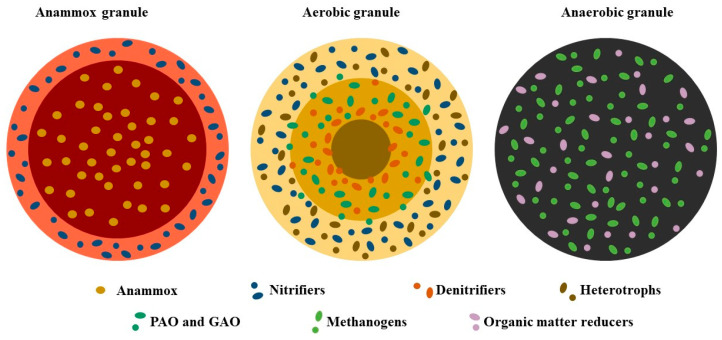
Schematic microbial distribution of the microorganisms in granular biofilms of aerobic, anaerobic, and autotrophic technologies. Color codes: aerobic granules (typical brown color); anaerobic granules (typical black color); anammox granules (typical reddish color of anammox granules). A change in the color of layers indicates a change in redox conditions; the darker the color, the lower the oxygen concentration.

The most widespread technology of WWT is the activated sludge process (AS), which is based on the continuous aeration of mixed-culture flocs since the systems are designed to degrade organic matter aerobically. Unfortunately, the increase in standard quality for wastewater discharge makes the exclusive use of AS unviable, requiring the implementation of available alternative technologies that offer an improvement from economic and environmental points of view [7]. One major disadvantage of AS is the generation of floc biomass because it advocates a loose structure, lower density, and poor settling capability, resulting in poorer effluent quality [8]; however, in the 1990s, AS with granular conformation was discovered, and its excellent performance opened up a new range of technologies for WWT. The granular sludge is a denser structure and consequently has better settling capabilities than flocs from AS. The compactness of the biomass reduces the volume of reactors, increases biomass retention, improves the resistance against shock loadings, and enhances the performance of the system. The systems based on granular biofilms also have an excellent nutrient-removal ability, as the spherical matrix with the embedded microorganisms limits the mass transfer of oxygen and nutrients from the external layer to the inner core, creating microenvironments in which nutrients can be efficiently removed (Figure 1). In addition, the behavior of the biofilm reduces the shock of toxic loading because it is diluted between the cells and the extracellular polymeric substances (EPSs) [9,10]. The EPSs have been reported to be essential in granular biofilm technologies for maintaining the structure of granules, even though there are some substances that are ubiquitous in aerobic, anaerobic, and autotrophic granules, such as sulfated glycosaminoglycans [9]. Together, this demonstrates that granules possess more favorable overall characteristics than flocs [8,11]. The granulation of biomass is employed for several technologies to encompass the degradation of diverse pollutants, depending mainly on the characteristics of the wastewater [12,13].

From the microbial ecology point of view in water and wastewater treatment systems, granular biomass is one of the most interesting ecosystems found yet. The gradient of substances from the outside to the inside of granular biomass makes possible the coexistence and synergic thriving of a multitude of different microorganisms That jointly allow the treatment of many different types of wastewaters. In addition, there is growing interest in the industrial field of wastewater treatment on the application of granular processes, making access to knowledge about microorganisms involved in biomass granulation and pollutant degradation in these systems a necessity for their future successful design, implementation, and operation at full scale.

Based on bioreactor and feed conditions, granular biomass could be roughly divided into three categories: aerobic granules (influent organic matter and oxygenated bioreactor), anaerobic granules (influent organic matter and unoxygenated bioreactor), and anammox granules (no influent organic matter and oxygenated bioreactor). Thus, this review is divided into these three main categories. Oxygenated granular biomass permits the presence of aerobic and anaerobic zones in the same granule, providing microecosystems that allow for the occurrence of different processes occurring in wastewater treatment, such as the removal of organic matter, nitrogen, and phosphorous. Therefore, sections of aerobic granules and anammox granules dive into these different microbial metabolisms, identifying the major ecological players based on recent literature. On the other hand, anaerobic granular sludge systems are used for the removal of organic matter and methane production only, thus leading to only two main microbial metabolisms: reduction of organic matter to acids and generation of methane. For this reason, the section on anaerobic granules identifies the effects that different conditions of the feed and the bioreactor have on the dominant microorganisms involved in these two features.

The literature search strategy considered the following keywords for each of the sections:

- Aerobic granules: “aerobic granular sludge” AND “microbial dynamic changes”, “enhanced biological phosphorus removal process”, “filamentous microorganisms”, “organic matter oxidizing microorganisms”, “granule formation”, “nitrification”, “denitrification”, and “bioproducts recovery”.
- Anaerobic granules: “upflow anaerobic sludge blanket” AND “pH”, “temperature”, “feed”, “alkalinity”, “toxic*”, “sulphate”, “sulfur”, “organic loading rate”, and “micronutrient”.
- Anammox granules: “anammox”, “granular anammox”, “anammox technology”, “*Candidatus* brocadiales”, “anammox technologies”, and “anaerobic ammonia oxidation”.

## 2. Microbial Ecology of Aerobic Granular Sludge

**Figure 2:**
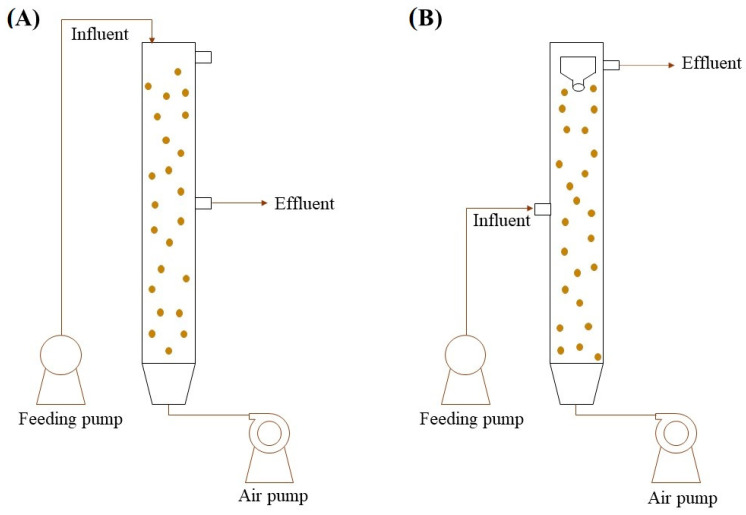
Schematic diagrams of aerobic granular sludge technology operated in sequential batch reactor (SBR) (**A**) and continuous flow reactor (CFR) (**B**). SBR reactor follows the next steps: fill with raw water from the top, then aeration period for pollutant degradation, followed by a settling period (granules decanting), and finally, the effluent is discarded (50% of the total volume). The configuration of the CFR reactor (front view) has a conical polyvinylchloride decanter placed in the water outlet zone for effluent discharge, and the reactor is fed by the half-height.

Aerobic treatments produce high-quality effluents in rapid processes; however, they require more energy for the needs of aeration and produce more sludge than anaerobic treatments [14]. AGS is a recent technology for WWT that reduces the disadvantages found in conventional aerobic technologies. In AGS, microorganisms are self-immobilized in dense and spherical biofilms with the assistance of EPSs [15]. The dense structure formed confers various advantages to the technology, and this high density provides a rapid separation of the biomass and aqueous phase [16]. In addition, EPSs allow the microbial population to withstand toxic substances and changing influents [10]. Moreover, there is a limitation on oxygen and mass transfer that creates differential conditions through the granule, resulting in diverse niches in which it is possible to establish different metabolisms. This permits the simultaneous removal of a wide range of contaminants, such as organic matter, nitrogen, phosphorus, or pharmaceuticals, among others [17]. These advantages are traduced in a compact technology that reduces construction and energy costs and produces high-quality effluents [16,18]. Traditionally, AGS has been operated in sequential batch reactors (SBRs). This mode of operation consists of the cycles of feeding, aeration, settling, and effluent discharge. SBRs permit a simpler selection of dense biomass; however, they have a complex construction, operation, and maintenance, and they cannot treat large flows efficiently [19]. For these reasons, there is currently an increasing interest in developing continuous-flow reactors (CFRs) for AGS systems, which includes the inherent challenge of selecting dense biomass and removing fluffy flocs that can disturb the proper functioning of the bioreactor (Figure 2).

### 2.1. Microbial Communities of Aerobic Granular Sludge

The microbial community present in AGS is affected by the inoculum, the influent characteristics, the bioreactor shape, the operational parameters, and the granule maturity [20,21,22].

#### Microorganisms Involved in Granule Formation

During the formation of granules from activated sludge inoculum, the microbial community changes and is selected according to the bioreactor and environmental conditions. Hydrodynamic forces, EPS secretion, filamentous microorganisms, and protozoa play an important role in the granulation process that starts with the contact between microorganisms, after which a three-dimensional structure is created, and finally, the granule is stratified [22,23].

EPS producers have been reported as an essential part of granule formation and stability; the more EPS producers in a granule, the denser and more stable it is [24,25,26]. EPSs are also involved in cell protection, impeding the direct contact of microorganisms with toxic substances [27,28,29]. This property allows aerobic granules to treat wastewater with diverse toxic substances and pollutants. Some EPS producers are *Flavobacterium*, *Acinetobacter*, *Thauera*, *Zoogloea*, *Devosia*, *Stenotrophomonas*, and *Rhodocyclus*, among others [27,30,31,32,33,34].

Several authors have reported the role of protozoa—especially stalked ciliates such as *Epistylis*—in granule formation due to their capacity to secrete EPSs and their control over suspended and peripheral bacteria, enhancing granular compaction [17,22,35,36]. Chan et al. [37] stated that in the absence of protozoa, the presence of *Candidatus* Accumulibacter was necessary for granulation. In addition, protozoa are also important in the removal of particulate matter and pathogen control [38].

Filamentous bacteria and fungal mycelium have been described as the backbone of aerobic granules due to their ability to increase the surface in which other microorganisms can be adsorbed, accelerating the granulation process [17,22,24]. Fungi could be more implied in granule formation when pH is low, whereas, at a higher pH, filamentous bacteria are more involved than fungi [32]. Some of these filamentous bacteria are members of the *Roseiflexus* and *Chloroflexi* genera [23,39]. Species of *Coriolus*, *Phanerochaete*, or *Aspergillus* are fungi that enhance granulation [40,41]. In addition, some filamentous bacteria such as *Thiothrix caldifontis* are able to consume acetate anaerobically, accumulate polyhydroxyalkanoates, and synthesize poly-P in the presence of sulfur compounds [42]; however, the instability of the system could be promoted by the overgrowth of filamentous microorganisms such as *Thiothrix*, *Sphaerotilus*, *Leptothrix* (bacteria), or *Geotrichum* (fungus) that can harm the settling properties of granules [26,35,43,44]. According to Aqeel et al. [45], the algae *Auxenochlorella* is associated with the instability of granular sludge, requiring the presence of the bacterium *Chitinophaga* to contrast its effect and maintain a stable formation. Although the overgrowth of filamentous microorganisms is easier in CFRs than in SBRs due to the better selection of dense biomass of the latter [46], reactor operations could control their growth and avoid the damage that they cause in granular density [42].

### 2.2. Microorganisms Involved in Pollutant Removal

A mature aerobic granule presents a microbial stratification according to the redox conditions. Thus, in a single aerobic granule, it is possible to find ordinary heterotrophic organisms (OHOs), ammonia-oxidizing bacteria (AOB), ammonia-oxidizing archaea (AOA), nitrite-oxidizing bacteria (NOB), complete ammonia oxidizers (comammox), denitrifying ordinary heterotrophic organisms (DOHOs), polyphosphate-accumulating organisms (PAOs), glycogen-accumulating organisms (GAOs), or even anaerobic ammonia oxidizers [22,47].

#### 2.2.1. Organic Compound Removal

Although a high number of microbial groups remove organic compounds, the main microbial groups involved in this reaction are OHOs and GAOs [47]. The *Xanthomonadaceae* family, the *Chloroflexi* class, and the *Flavobacterium* genus belong to OHOs, whereas *Accumulibacter* and *Defluviicoccus* genera are GAO representatives [47]. Fungi from the *Ascomycota* phylum, such as *Scopulariopsis* or *Penicillium*, have been reported as organic matter degraders [26,48]. In urban wastewater, Burzio et al. [49] stated that *Rubrivivax* was the most abundant genus for COD removal. For pig slurry treatment, *Comammonas*, *Zoogloea*, and *Thauera* are the main organic-matter-oxidizing bacteria present [50]. Rosa-Masegosa et al. [51] highlighted the prominence of *Flavobacterium*, *Methylophilus*, *Stenotrophomonas*, and *Thauera* for treating sulfur amino-acid-rich wastewater. *Pseudomonas* and *Thiothrix* have been reported to grow in the presence of sulfamethoxazole and to play a role in removing it [52,53]. In the presence of carbamazepine, diclofenac, naproxen, and trimethoprim, a proliferation of *Flavobacterium* and *Leadbetterella* was observed [54]. Phenolic compounds are also degraded by microorganisms such as *Acinetobacter*, which is implied in the removal of *p*-nitrophenol [55]. *Arenimonas*, *Lampropedia,* and members of the *Xanthobacteraceae* family are involved in caffeic, hydroxybenzoic, and protocatechuic acid removal [56]. In addition, *Sphingobium* has a role in 4-chlorophenol degradation [57], and *Ralstonia*, *Sphingomonas*, and *Pusillimonas* have been reported to remove bisphenol A [58].

#### 2.2.2. Nitrogen Removal

The removal of nitrogen (N) involves several microbial metabolisms. Simultaneous nitrification and denitrification is possible to execute in AGS [17]. AOB and NOB, which are implied in the nitrification process, can be found in the outer layer of granules [17,22]. *Niabella* and *Nitrosomonas* are AOB and transform ammonium to nitrite, whereas NOB such as *Candidatus* Nitrotoga and *Nitrobacter* are responsible for nitrite oxidation to nitrate [49,52,59,60]. Some bacteria are able to complete the two steps of nitrification in a process called complete ammonia oxidation (comammox), whose main representative in AGS is *Nitrospira* [49]. The denitrification process can be carried out under anaerobic and aerobic conditions; thus, denitrifying bacteria that reduce nitrate to nitrogen gas could be present in the inner and outer layers. Some of these microorganisms are *Thauera*, *Zoogloea*, *Pseudomonas*, *Paracoccus*, *Dokdonella*, and *Spirosoma*, among others [26,59,60]. Strains of *Hydrogenophaga* and *Diaphorobacter* genera have been reported in heterotrophic nitrification and aerobic denitrification [59,60]. The ammonium-removal process is more efficient than nitrification and denitrification because the oxidation of nitrite to nitrate and the posterior reduction to nitrite is not necessary; thus, it requires less oxygen and electron donors. To achieve this process, it is essential to reduce the growth of NOB. This is achieved by limiting the dissolved oxygen in bioreactors, maintaining a temperature higher than 25 °C and a low sludge-retention time [17]. In addition, an aerobic granule with an anammox core has been reported [19]. In fungi, the phylum *Ascomycota* has been recognized for its capacity for nitrogen removal [26]. Some studies have reported that algal–bacterial granular sludge can increase nutrient removal and be energy saving [23,61]. *Nitzschia*, *Chlorella*, *Neodesmus*, *Scenedesmus*, *Leptolyngbya*, *Oxyphotobacteria*, *Prochlorotrix*, and *Auxenochlorella* are algae found in AGS [17,45,61,62]. High-salinity conditions produce instability in granules and affect nutrient removal; however, it has been reported that, under these conditions, algal–bacterial granular sludge achieved higher stability and better nutrient reduction than bacterial granular sludge [62].

#### 2.2.3. Phosphorus Removal

PAOs are the main microorganisms responsible for phosphorus (P) removal in AGS by means of an enhanced biological phosphorus removal (EBPR) process [17,63]. The EBPR process is the biological mode of P removal; thus, it is a better alternative than chemical P removal [64]. The EBPR process consists of alternating anaerobic and aerobic phases. During anaerobic conditions, PAOs hydrolyze poly-P to obtain energy, whereas, in aerobic conditions, they uptake and accumulate the excess P in the form of poly-P [23]. In addition, EPS plays an important role in P removal because poly-P is also accumulated in EPS bound to cells [17]. Another mechanism for P removal is the precipitation with Ca or Mg [23,34,65]. PAOs and GAOs are considered slow-growing microorganisms, and they can be found in the outer and inner layers of dense granules with anoxic microenvironments [17,22,34]. Typical PAOs in AGS are *Rhodocyclus*, *Dechloromonas*, *Tetrasphaera*, *Flavobacterium*, *Corynebacterium*, *Pseudomonas*, *Acinetobacter*, and *Candidatus* Microthrix parvicella, although the main PAO is *Candidatus* Accumulibacter phosphatis [20,34,36,63,66]. The main genera representatives of GAO are *Candidatus* Competibacter, followed by *Candidatus* Contendobacter, *Propionivibrio*, and *Defluviicoccus* [22,36,37,39,66]. Temperature, pH, salt concentration, wastewater composition, aeration strategy, and SBR cycle are parameters that usually affect the dynamics of PAOs and GAOs; thus, P removal by AGS [17,23]. Some authors affirmed that an increase in salt concentration produced a decrease in P removal due to the loss of PAOs associated with the increase in GAOs [17,23]. It is also known that there is competition between PAOs and GAOs for carbon sources [34,65]. High temperature (20–30 °C) generates an increase in GAOs and, thus, a decrease in PAOs [67]. Current investigations are focused on how to reduce GAO activity to maintain P removal [65]. Filamentous bacteria such as *Thiothrix* compete against PAOs and GAOs, especially at a high COD. A high sludge-retention time and a high COD usually produce a decrease in PAOs in favor of GAOs and filamentous bacteria [31,63]. According to Li et al. [63], *Candidatus* Accumulibacter phosphatis is more competitive and is the first PAO that is recovered when filamentous bacteria are limited. PAOs are favored by propionate, acetate, succinate, and endoleic acid substrates [17,23,31], while glucose, acetate, and sometimes propionate are substrates that increase the abundance of GAOs [31,65,68,69]. De Sousa Rollemberg et al. [69] affirmed that under the combination of propionate as the only electron donor and nitrite as the unique electron acceptor, a washout of GAOs occurs. The enrichment in PAOs creates denser granules with better settleability and more stability [22,23,27,39]. This enhances the selection of PAO granules in CFR operation [70]. The main phosphorous recovery is carried out by PAOs, and the proliferation of GAOs reduces the available niche of PAOs to compete (Figure 1), resulting in decreased phosphorus removal and a deterioration in the performance of biotechnological approaches [71].

In AGS, it is also possible to find denitrifying PAOs (DPAOs) and denitrifying GAOs (DGAOs). DPAOs are microorganisms capable of simultaneous denitrification and phosphate removal, and sometimes, these microorganisms can store organic carbon as PHA and use it in the denitrification process as an electron source. DPAOs are energetically more efficient because they need less aeration, and they remove COD, N, and P [17]. For these reasons, the conditions promoting DPAOs should be detected and implemented in WWT. Some of these microorganisms are unclassified *Candidatus* Accumulibacter, *Thauera*, and *Dechloromonas* strains [17,37]. In addition, some authors have identified combinations of microorganisms that can achieve high levels of denitrification and P removal, such as *Candidatus* Competibacter with *Candidatus* Accumulibacter, *Xhanthomonadales*, and *Tetrasphaera*, or *Candidatus* Accumulibacter, *Nitrosomonas*, and *Nitrospira* [34].

### 2.3. Microorganisms Involved in Valuable Bioproduct Recovery from WWT

Tavares Ferreira et al. [72] compiled a list of the resources that AGS could generate: water reuse, phosphorus, alginate-like exopolysaccharides (ALEs), polyhydroxyalkanoates (PHA), and tryptophan, among others. It has been shown that AGS not only generates a high-quality effluent requiring fewer resources, but it also produces biotechnologically interesting substances simultaneously. The isolation of these products represents a reduction in operational expenditure and promotes the circular economy [72]. The recovery and application of interesting substances from AGS are being studied [31]. Cydzik-Kwiatkowska et al. [73] have extracted ALEs from a full-scale AGS, and they have used it for cadmium adsorption. The AGS microbial community is involved in these activities. PAO are the main microorganisms responsible for P recovery that can be used in agriculture. ALEs are a polymer applied in the textile, chemical, and paper industries as surface-coating material due to their water-resistant and flame-retardant properties. In addition, they can be applied in soils for water retention. *Algae*, *Pseudomonas*, and *Azotobacter* are the main microorganisms implicated in ALE production [74]. On the other hand, PHA is a polyester used in the biofuels, packaging, and ink industries and the biomedicine sectors. *Acidovorax*, *Comamonas*, *Paracoccus*, and *Thauera* are some of the responsible microorganisms capable of PHA accumulation [75]. *Thauera* and *Paracoccus* are also important genera in the production of tryptophan, which is a hydrophobic amino acid employed in the agriculture, chemical, and pharmaceutical sectors [72]. *Streptococcus*, *Escherichia*, and *Pasteurella* have been reported as producing glycosaminoglycans, which are a useful polysaccharide in the biomedical, pharmaceutical, and food industries [76]. Xanthan polysaccharide is produced by *Xanthomonas* and is used in the pharmaceutical and food industries [77].

## 3. Microbial Ecology of Anaerobic Granular Sludge

Anaerobic WWT technologies have advantages over aerobic technologies, such as their lower sludge generation, lower space requirements, and higher energy efficiency [14]. It has been estimated that anaerobic treatments save up to 1 kWh per Kg of COD removed compared to activated sludge systems [78]. Some anaerobic WWT technologies that are widely used in the field today spontaneously form granular biomass, among which the up-flow anaerobic sludge blanket (UASB) is the most prominent.

**Figure 3:**
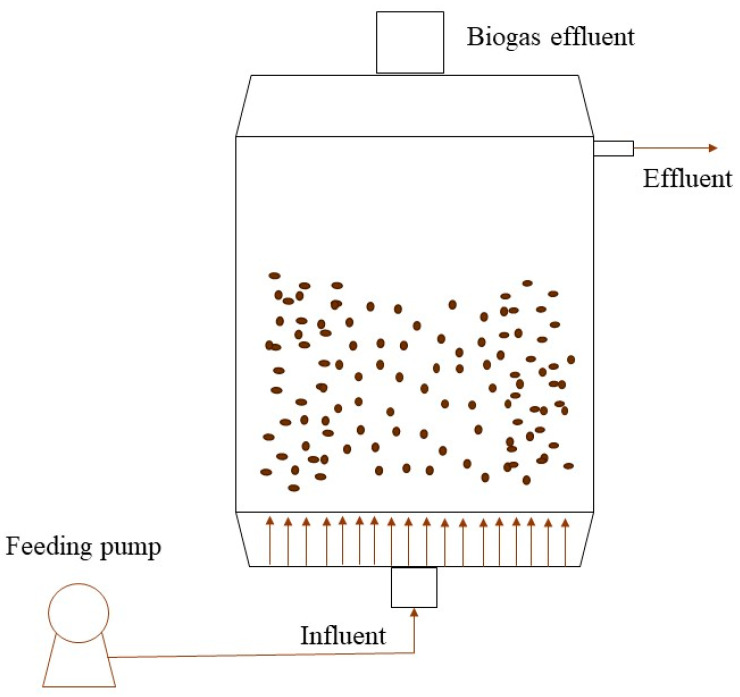
Diagram of a UASB reactor.

The UASB technology was first proposed in the 1970s by Lettinga and co-workers [79] and has been used for the treatment of industrial wastewater since then, recently receiving a spike in attention [80]. The main function of the UASB consists of an up-flow reactor in which the influent is forced to pass through a sludge blanket, which develops all biochemical reactions needed for the treatment of the feed (Figure 3). The effluent and generated gases are collected upward to the sludge blanket [81].

As mentioned above, one of the most important advantages of the UASB over other WWT technologies is its superior energy efficiency. UASB reactors generate methane, which provides energy recovery along with the treatment of wastewater [82]. Moreover, UASB reactors have low sludge production, minimizing the costs involved in its treatment and the inconveniences related to their handling and disposal [83]. On the other hand, the UASB technology has some disadvantages that need to be addressed, such as slow start-ups (mitigated by inoculation with granular biomass) and the required treatment of effluent for the removal of nutrients and pathogens [83]. Indeed, substantial quantities of pathogenic organisms such as *Cryptosporidium* and *Giardia* have been identified in UASB effluents [84], which could lead to human and environmental health concerns.

### 3.1. Microbiota of UASB Technology

Research on the microbiota developing in UASB systems aims to elucidate the link between certain microbial phylotypes and the removal of target pollutants, as well as elucidate the effect that different operational conditions have over it. An important aspect of the UASB system is the spontaneous granulation phenomenon of its biomass, which is not yet fully understood at the mechanistic levels [80]. In any case, the UASB operates under anaerobic conditions for the treatment of organic matter, which involves the reduction of organic matter up to volatile fatty acids and their consumption for the formation of methane and other gases. Thus, the microbial communities in UASB systems resemble those of the anaerobic digestion systems, presenting methanogenic archaea that are syntrophic with bacterial members.

There are limited studies of non-archaeal, non-bacterial microorganisms (for example, Eukarya) in UASB systems, given that they do not play a major role in the functioning of the technology; however, it has been found that several genera are usually present, such as *Epistylis*, *Telotrochidium*, *Tetrahymena* and *Vorticella* (phylum *Ciliophora*), *Phalansterium* and *Saccamoeba* (phylum *Amoebozoa*), *Cercomonas*, *Heteromita*, and *Rhogostoma* (phylum *Cercozoa*), and *Protoperidinium* (phylum *Dinoflagellata*) [85]. Moreover, parasitic *Cryptosporidium* (phylum *Apicomplexa*) has been detected in UASB effluents [84], which raises concerns about the safe handling of UASB-treated water streams.

A revision of recent literature suggested that the microbial communities in UASB systems could be separated into methanogens and bacterial reducers of organic matter and/or other chemical compounds. For the methanogens, the more prevalent phylotypes are classified as *Methanosaeta* and *Methanomicrobium*. These are present in most cases of UASB systems regardless of the feed used; therefore, these could be thought of as core microbes of this technology. The difference between the two consists of the higher metabolic flexibility of *Methanomicrobium*, as it can also thrive on hydrogen, while *Methanosaeta* solely uses acids for growth. With respect to bacterial reducers, these are more dynamic than methanogens and change with the feed used and operational conditions.

### 3.2. Effects of Feed: Nature of Feed, Time of Feeding, and Pre-Treatment of Feeding

The myriad of different feeds treated with UASB technology generate a vast diversity of microbiota that, in most cases, appear to be unique for the feed used. Thus, the diversity of microorganisms that have been found in UASB systems is quite large and case specific. Important cases found in recent literature are summarized below.

An experiment using UASB technology for the treatment of conventional and vacuum toilet flushing showed significant differences in the microbial communities developing in the UASB systems. Differences were caused by higher organic matter and ammonium content in vacuum toilet flushing in comparison with conventional toilet flushing. For conventional toilet flushing, the dominant methanogen was *Methanolinea*, while for vacuum toilet flushing, the dominant methanogen was *Methanogenium* [86]. Conventional toilet flushing UASB supported the growth of the sulfate-reducing bacteria *Desulfobacteraceae*, *Desulfobulbaceae*, and *Desulfomicrobiaceae*, and the bacterial families *Campylobacteraceae*, *Rhodocyclacae*, and *Pseudanabaenaceae*. On the other hand, the vacuum toilet-flushing UASB supported the growth of the *Fibrobacteraceae* and *Porphyromonadaceae* families and the *Clostridiales* order [86].

Time of feeding also has an impact on function and the microbial communities in UASB systems. For instance, it has been reported that a change from continuous (1 g COD L^−1^ day^−1^) to semi-continuous (2 g COD·L^−1^ day^−1^ for 12 h or 0 g COD·L^−1^ day^−1^ for 12 h) feeding in UASB reactors caused the decrease in acetoclastic *Methanosaeta* and favored the growth of acetoclastic and hydrogenotrophic *Methanosarcina* [87]. The dominant bacterial phylotype, *Sporomusa*, shared the dominance with *Leucobacter*, which followed the same trend over time. Pre-acidification of feed also plays an important role in the microbiome of UASB reactors. For instance, pre-acidified and non-pre-acidified UASB reactors treating starch wastewater showed significant differences in microbial phylotypes involved in the processes, with a higher abundance of *Methanosaeta* than *Methanomicrobium* if pre-acidification was present and the contrary in its absence [88]. The dominance of *Methanosaeta* over *Methanomicrobium* in the pre-acidification reactor was also linked to the higher operational stability of the system.

The treatment of hydrolyzed wheat straw and lucerne in UASB resulted in the dominance of the bacterial phyla *Firmicutes* (dominated by order *Rhizobiales*) and *Synergistetes* (dominated by genus *Aminivibrio*), along with a minor representation of *Bacteroidetes*, *Chloroflexi*, *Proteobacteria*, or *Spirochaetes*. For the *Archaea* domain, the dominant phylum was *Euryarchaeota* [89]. It was found that the main drivers of the microbial community were the concentrations of acetic, organics, and propionic acids, followed by organic loading rate (OLR) and influent COD for this feed. Both *Rhizobiales* and *Aminivibrio* were negatively correlated with organic acids. The dominant archaeal phylotypes found were *Methanosaeta* and *Methanobacterium*, transitioning in the systems due to a higher tolerance to acidification of the former with respect to the latter [89].

### 3.3. Effect of Organic Loading Rate

The OLR is an operational parameter that has been found to drastically impact the microbiome of UASB reactors. For example, a UASB system treating *Chlorella vulgaris* biomass at different OLRs suffered a decrease in methanogenic microbiota (mainly *Methanosaeta* archaeon) and the bacterial phylum, *Chloroflexi*, and an increase in hydrolytic and fermentative microbiota (mainly bacterial phylum *Firmicutes* and *Methanobacterium* archaeon) at increasing OLRs [90]. Increasing the OLR in UASB reactors treating monosodium glutamate wastewater showed a shift in the methanogenic archaea community from the dominance of *Methanosaeta* and *Methanobacterium* at a low OLR to the dominance of *Methanosarcina* and *Methanobacterium* at a high OLR [91]. Increasing the OLR was also shown to promote the growth of the *Firmicutes* phylum, while the *Actinobacteria* phylum showed the opposite trend.

### 3.4. Effect of Operational Parameters pH, Temperature, and Alkalinity

The operational pH severely affects the microbial communities of UASB and, thus, its performance. It was found that a decrease in influent pH from 7.0 to 5.0 in UASB-treated sugar refinery wastewater led to a decrease in *Methanosaeta* abundance coupled with an increase in *Methanospirillum*, *Methanosarcina*, and *Methanobacterium* as pH values dropped [92]; however, *Methanosaeta* was the dominant methanogen at all pH conditions tested. In the same fashion, the bacterial genera *Prevotella*, *Streptococcus*, and *Acidaminicoccus* decreased with pH, while the bacterial genera *Megasphera*, *Butyricicoccus*, *Lactococcus*, *Parabacteroides*, and *Desulfovibrio* increased with decreasing pH.

The operational temperature of UASB systems also affects their microbial communities, promoting the growth of psychrophilic, mesophilic, or thermophilic microorganisms, depending on the case. Reports of seasonal variations in microbial communities in UASB reactors treating domestic sewage in southern Brazil showed the prevalence of psychrophilic–mesophilic *Arcobacter*, *Trichococcus*, *Thauera*, and *Zoogloea* bacteria, among others, during the winter [93]. On the other hand, summer temperatures promoted the growth of *Methanolinea* methanogen and *Caldisericum* and *Desulforhabdus* bacteria. Similar operations of UASB reactors at different temperatures have reported that different microorganisms are more favored under certain conditions; for example, *Methanosaeta* seems to prefer room temperature conditions, while *Methanobacterium* seems to prefer mesophilic temperatures [94].

Feed alkalinity has also been shown to affect the microbiome of UASB reactors. Decreasing feed alkalinity (from 2800 to 700 mg·L^−^^1^) in UASB-treated synthetic wastewater affected the populations of the methanogens *Methanosaeta* and *Methanobacterium*, which were displaced by *Methanolinea* and *Methanoregula* [95]. In the case of bacterial members, higher alkalinity selects for *Kluyvera*, while decreasing alkalinity selects for *Clostridium*, *Bacteroides*, *Mesotoga*, or *Longilinea*, among others.

### 3.5. Effect of Sulfate

Unquestionably, the nature and characterization of the treated wastewater substantially modify the inherent microbial ecology that would be described in cases of urban wastewater with organic matter, nitrogen, and phosphorus. This is why industrial water treatments promote the growth of microorganisms capable of competing in the presence of contaminants that differ from N and P. There are contrasting opinions on the effect of sulfate on the microbial community structure of anaerobic WWT systems. While some evidence shows that the ratio of COD/SO_4_^2−^ has an impact on the community structure of *Archaea* and *Bacteria* in these systems, other theories support that only the COD and not the COD/SO_4_^2−^ ratio affects the diversity and community structure of these microorganisms in anaerobic treatment systems [96,97,98]. In any case, there is current interest in the scientific community in observing the effect of sulfate on UASB reactors. The continuous feeding of sulfate to UASB systems was found to promote the growth of the sulfate-reducing bacteria *Desulfovibrio*, along with *Propionispora* and *Syntrophobacter* [99]. At the same time, the community of methanogenic archaea in the reactor, mainly represented by *Methanosaeta*, decreased with continuous sulfate feeding, leading to the UASB losing methanogenic capacity in favor of sulfidogenic capacity. The addition of sulfate to a UASB system treating starch wastewater was shown to promote the proliferation of *Methanosaeta* and *Methanobacterium* and to cause a shift in the bacterial community dominated by *Levilinea* and *Propionivibrio* to a system dominated by *Ruminococcus* and *Desulfovibrio* [100]. Linked to the increase in the relative abundance of *Methanosaeta*, biogas production increased with the addition of sulfate to the UASB.

### 3.6. Effect of Micronutrients and Toxic Pollutants

The addition of micronutrients such as copper to the UASB feed is capable of improving its removal performance, as well as changing its microbiota. The addition of copper as CuCl_2_ and CuSO_4_ to UASB systems treating food waste and domestic wastewater resulted in a decrease in *Methanobacterium* and an increase in *Methanolinea*, while the population of *Methanosaeta* remained unaffected [101]. The *Proteobacteria* and *Chloroflexi* phyla decreased substantially after copper addition, along with an increase in the *Bacteroidetes*, *Thermotogae*, *Synergistetes*, and *Spirochaetes* phyla. The addition of copper promoted the growth of *Acetitomaculum*, *Mesotoga*, and *Gelria* bacteria, but many dominant bacterial genera are susceptible to copper, and the microbial communities changed at different copper concentrations (from 20 mg·L^−^^1^) [102].

UASB systems treating selenate from wastewater showed differential microbial communities in the presence of cadmium and zinc [103]. In this sense, the addition of cadmium promoted the growth of *Desulfovibrio*, *Macellibacteroides*, and *Tyzzerella*, while the addition of zinc promoted the growth of *Pseudomonas*, *Desulfovibrio*, and *Enterobacteriaceae* family members. *Methanosaeta* was the dominant methanogen under all conditions tested.

The addition of ferrihydrate to a UASB treating synthetic sulfate wastewater showed significant differences in its microbiota with respect to a control UASB without ferrihydrite [104]. With the continuous addition of ferrihydrite, the dominant bacterial phylotypes were *Desufovibrio*, *Clostridium*, *Veillonella*, and *Desuldomicrobium*. On the other hand, the dominant bacterial phylotype in the control reactor was *Aminicenantes*. With respect to archaeal members, however, the addition of ferrihydrite did not seem to have a significant effect, with both reactors being dominated by the methanogen *Methanothrix*.

UASB technology has been proven to efficiently remove toxic pollutants from wastewater under extreme environmental and operational conditions. Research conducted on the removal of guar under very high salinities (up to 10 Kg·L^−^^1^ NaCl) in UASB showed a high removal capacity [105]. Without salinity, the dominant bacterial players reported were *Bacteroides*, *Prolixibacter*, and *Pelolinea*. Increasing salinity supported the development of *Proteiniphilum* and *Aminiphilum* at medium levels and of *Mesotoga* and *Lentimicrobium* at higher levels. On the contrary, increasing salinity levels reduced the relative abundance of the methanogenic archaea community, dominated by *Methanosaeta*.

Another example of a toxic pollutant that can be removed by UASB is azo dyes. A micro-aerated UASB was able to provide the successful removal performance of the azo dye, Direct Black 22 [106]. Its microbiota was composed of *Methanosaeta* and *Methanobacterium* methanogens along with the fermentative bacteria *Trichococcus*, *Clostridium*, *Bacteroides*, or *Fusobacterium*, in addition to *Syntrophus* as a syntrophic bacterium and the sulfate-reducing bacteria *Desulfobulbus*, *Desulfovibrio*, *Desulfotomaculum*, and *Desulfomicrobium*. An increase in salinity during treatment promoted the growth of *Trichococcus* and *Brevundimonas* bacteria.

The addition of antimicrobial compounds can also affect the microbial communities in UASB reactors. The addition of the antibiotics tetracycline, erythromycin, and sulfamethoxazole to a UASB promoted a decrease in the relative abundance of *Bacteroidetes* phylum in comparison with the control and an increase in the relative abundance of *Halobacterota* and *Euryarchaeota* in comparison with the control [107]. *Methanobacterium* showed higher relative abundance in the control bioreactor, while *Methanosaeta* was favored under antibiotic pressure.

## 4. Microbial Ecology of Autotrophic Granules

Autotrophic granular technologies have emerged as a sustainable approach that is superior to the conventional nitrification/denitrification process due to savings in energy cost, the possibility of resource recovery, the lack of requirements for an external carbon source, sludge disposal, and being an eco-friendly system [108]. These technologies are based on the anaerobic ammonium oxidation (anammox) process, which is well-known for treating nitrogen-rich wastewater, such as the treatment of sludge-digester supernatant.

### 4.1. Autotrophic Nitrogen Removal Process

This process occurs under two diverse sets of oxygen conditions; thus, the application of this technology has been developed in a single chamber such as completely autotrophic nitrogen-removal over nitrite (CANON), ANITA Mox, Cleargreen, TERRAMOX, DEMON, and Anammox SBR systems, or in two diverse, consecutive chambers based on partial nitrification-anammox (SHARON-anammox). The process based on granules has been given different names: CANON [109], oxygen-limited autotrophic nitrification–denitrification (OLAND) [110], and de-ammonification [111] processes. The technology will be referred to as CANON in this manuscript for being the best known.

**Figure 4:**
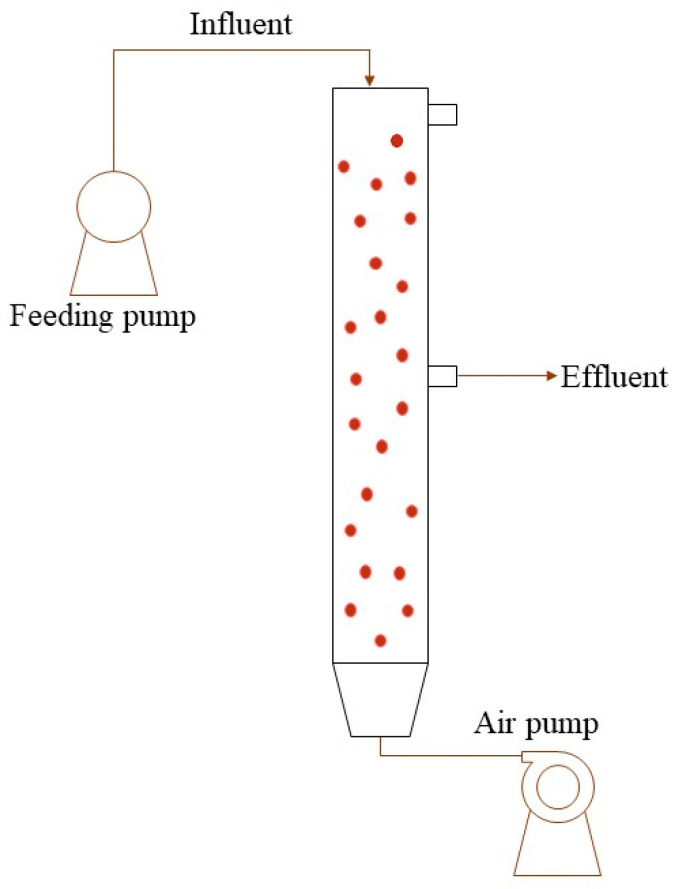
Schematic diagram of CANON in an SBR. SBR reactor follows the next steps: fill with raw water from the top, then aeration period for pollutant degradation, followed by a settling period and the effluent discards.

CANON utilizes oxygen-limited conditions to perform simultaneous partial nitrification and anaerobic ammonium oxidation in a single reactor, which is achieved by nitrifying microorganisms and anaerobic ammonium-oxidizing bacteria (AnAOB), respectively [108], that coexist within the reactor due to the configuration of granular biomass (Figure 4). Compared to the traditional nitrification–denitrification process, it consumes 63% less aeration due to the strong restriction of dissolved oxygen and nearly 100% less carbon, making it possible to achieve energetic self-sufficiency in WWT plants [11]. The generated biomass caused by the hydraulic shear force produced granules with sizes of >200 μm. This has become an interesting hotspot because it improves the settling properties (70–150 m·h^−^^1^), resulting in a longer sludge-retention time, and is a feasible choice for the CANON process [112].

### 4.2. Aerobic Ammonia Oxidation

To date, AOB include five genera *Nitrosospira*, *Nitrosococcus*, *Nitrosolobus*, *Nitrosomonas*, and *Nitrosovibrio* [113,114]. The most typical AOB described in anammox systems is *Nitrosomonas*, which can survive in anoxic or anaerobic conditions [113,115,116], while the rest of the *Betaproteobacteria* are widely distributed in natural environments [113]. *Nitrosospira* is also a competitive AOB in systems with low dissolved oxygen and nitrogen load (below 0.5 mg·L^−^^1^) [117]. The authors of this study also pointed out that its contribution was five times over that of *Nitrosomonas* [117], although it is common to find several species within *Nitrosomonas*, such as *N. europaea*, *N. eutropha*, and *N. oligotropha*. *N. europaea* is the best-characterized ammonia-oxidizing bacteria to date [118,119]. In fact, one of the most important concerns currently is related to antibiotic-resistant genes affecting nitrification, as recent studies have pointed out the decrease in the ammonia removal capability of *N. europaea* through conjugation transfer [120]. *Nitrosomonas* release nitric oxide (NO) either during NH_2_OH oxidation at atmospheric oxygen levels or via nitrite reduction under oxygen-limited conditions [121]. It has been confirmed that NO is an obligate bacterial nitrification intermediate produced by hydroxylamine oxidoreductase (*hao*) or nitrite reductase (*nir*) [122].

Additionally, the wastewater characteristics modify the abundance of dominant AOB. In some studies, it is pointed out that under extremely high nitrogen conditions, *N. eutropha* outcompete *N. europeae* [114]. Moreover, the addition of industrial effluents such as landfill leachates had an important effect on the morphologic structure of the AOB community and, consequently, changes in the spatial distribution of the flocs [123]. Rodriguez-Sanchez et al. [124] reported that the antibiotic presence was to the detriment of *N. europeae*, while this ecological niche could be taken by *N. eutropha*, but long-term operation worsened the situation of AOB. Results of both studies corroborated the hypothesis that in the presence of high nitrogen concentrations and any organic carbon, *N. europeae* in partial nitrification systems could be an r-strategist species in the community [125]. On the contrary, *Nitrosospira* is usually detected in anammox technologies, but their abundance used to be much lower than that of *Nitrosomonas*; therefore, *Nitrosospira* is considered a k-strategist genus in anammox systems [117,125]. In fact, the r/K strategists were observed in the research carried out by Gonzalez-Martinez et al. [126], in which the drop in temperature (<25 °C) promoted the growth of *Nitrosospira* and the decrease in *Nitrosomonas* and *Nitrosobacter*. Moreover, in CANON systems, bacteria involved in ammonia removal, such as *Prosthecobacter*, were found at a higher relative abundance than *Nitrosomonas* or *Nitrosospira* because they could find an optimal niche to proliferate due to the appearance of organic matter caused by temperature selection, subsequent bacterial death, and the presence of (NH_4_)_2_SO_4_ as an ammonium source [126].

Not only *Bacteria* have been detected in anammox systems as prokaryotic microorganisms. Some *Archaea* also harbor archaeal ammonia monooxygenase genes to encode enzymes for catalyzing ammonia oxidization [127,128]. AOA are a heterogeneous group that play an essential role in global biogeochemical cycles and are widely distributed in diverse environments [129]. To date, AOA includes eight major clusters affiliated with *Nitrosopelagicus*: *Nitrosopumilus*, *Nitrosotalea*, *Nitrosocosmicus*, *Nitrososphaera*, *Nitrosocaldus*, *Nitrosoarchaeum*, and *Nitrosotenuis*. *Nitrosopumilus martimus* and *Nitrososphaera viennensis* were the two most studied *Thaumarchaeota* species, with the goal of understanding their implication in the nitrogen cycle [129]. *Nitrososphaera* spp. is one of the dominant AOA in anammox systems, which could be enriched with intermittent aeration [130,131], while other authors found *Nitrosopumilus maritimus* to be one of the dominant AOA species in the general treatment trains of wastewater [132].

AOA possess advantages over AOB because they need lower half-saturation constant values for dissolved oxygen and ammonia in comparison with AOB. In fact, that allows them to adapt to extremely low electron donor/acceptor conditions, which indicates that AOA outcompete AOB for ammonia and oxygen under low ammonium and DO conditions [133]. The affinity for an electron donor of *Nitrosopumilus maritimus* is higher than that of *N. europaea* [127]; however, in anammox systems in which the ammonia concentration was high, the AOA community was negatively correlated with it [134]. Some organic products such as succinate, pyruvate, and malate could accelerate the growth of this group because they act by detoxifying hydrogen peroxide generated in the ammonia oxidation metabolism [135]. The *Nitrosocosmicus* genus could produce EPSs that encourage the dense structure of granular aggregates in CANON technology, which also offers higher resistance to adverse conditions [136,137] and can be consumed by heterotrophic bacteria as a substrate. The results obtained by several studies changed a conventional perception that AOB are the only or dominant group of nitrifiers in the WWT process [133].

### 4.3. Anaerobic Ammonia Oxidation

Anammox metabolism was discovered for the first time in a denitrification fluidized bed reactor used for WWT by Mulder et al. [138]. This group of bacteria belongs to the *Planctomycetes* order [139], whose metabolism is strictly anaerobic and autotrophic. Anammox bacteria are characterized by a compartmentalized cell architecture featuring a central membrane-bound compartment known as the “anammoxosome”. The anammoxosome membrane contains ladderane lipids operating as a strong physical barrier to limit the transference diffusion of metabolites [140]. This structure occurs in the cells from the production of highly toxic compounds as a result of their own metabolism. The reaction is based on metabolizing ammonia and nitrite at a 1:1 ratio to generate dinitrogen gas. The first step of the anammox reaction is to reduce nitrite to nitric oxide by nitrite oxidoreductase (*nir*). Next, ammonium and nitric oxide are combined to produce hydrazine by hydrazine synthase (*hsz*). Finally, hydrazine is dehydrogenated by hydrazine oxidoreductase (*hzo*) to dinitrogen gas [141]. Hydrazine is toxic to all microorganisms if it is accumulated inside for long periods, even anammox bacteria. The unusual metabolic pathways of anammox bacteria produce extensive concerns in the microbial nitrogen cycle and biological WWT [142].

An important concern regarding anammox for their application in biotechnological approaches is the slow growth and having a specific velocity of maximum growth (µmax) of 0.065 d^−^^1^ and a replication time of 11–22 days [143], supposing a critical point regarding the enrichment of anammox bacteria in bioreactors. The growth is modulated by the low rate of ammonia consumption (0.4 µmol NH_4_^+^ mg protein^−^^1^ min^−^^1^), so long-term operation is needed when inoculation is conducted with an unspecific sludge. Nowadays, the implementation of this process is conducted in a single-chamber reactor based on biofilms, allowing the coexistence of aerobic and anaerobic niches. In the external layer of the biofilm, the dissolved oxygen allows for the oxidation of ammonia to nitrite under aerobic conditions carried out by AOB and AOA, while in the internal layers, the anoxic conditions promoted a quicker method for nitrogen gas production. The optimal temperature for the growth and activity of anammox bacteria is in the range of 35–40 °C, but these phylotypes can habituate in natural ecosystems at ultra-low temperatures (from −5 to 4 °C), as is the majority of biospheres [144] and also at thermophilic conditions (50–80 °C) [142,145].

Six genera of anammox bacteria have been currently discovered including *Candidatus* Jettenia, *Candidatus* Brocadia, *Candidatus* Anammoxoglobus, *Candidatus* Anammoximicrobium, *Candidatus* Kuenenia, and *Candidatus* Scalindua, all belonging to the family *Candidatus* (Ca.) Brocadiaceae of the order Ca. Brocadiales is in the *Plantomycetes* phylum [146].

*Candidatus* Brocadia and *Candidatus* Kuenenia are the main AnAOB detected in anammox systems, but their relative abundance used to be in the range of 0.2–20% [117]; however, other authors have reported that Ca. Brocadia is the predominant AnAOB member in CANON systems at low temperatures or under shock loading [13,126,147]. Particularly, the biodiversity of the genus Ca. Scalindua might have been largely underestimated, and only a few studies have identified it in CANON technology [148,149]. Interestingly, Ca. Scalindua was detected in anammox systems based on biofilms attached to the carrier or suspended growth [150,151]. Ca. Jettenia is known for its resistance to high-salinity concentrations, and its importance is reported in granular anammox systems treating wastewater with concentrations lower than 15 g·L^−^^1^ [152].

The anammox bacteria are usually highly sensitive to operational conditions such as nitrite concentration, high salinity, temperature, sulfides, toxic metal elements, and toxic organic compounds, whose changes can induce the inhibition of anammox growth. For example, relative to hydraulic retention time (HRT), Ca. Jettenia could not grow under short HRT [117]. At low temperatures, anammox granules lost diversity and Ca. Brocadia was promoted [126]. In addition, Ca. Brocadia is highly resistant to the stress exerted by the presence of organic loads, maintaining high nitrogen activity up to 300 mg O_2_·L^−^^1^ of COD [153]. Studies on the tolerance against the presence of low-concentration antibiotics in the wastewater showed that Ca. Kuenenia is a high relative-abundance phylotype for long-term operation [136].

### 4.4. Nitrite-Oxidizing Bacteria and Denitrifier Bacteria

The restriction of NOB and denitrifying bacteria is critical for the profitable application of granular anammox technology because the proliferation of these groups can outcompete the AnAOB [125]. For this reason, the monitoring of the NH_4_^+^/NO_2_^−^ ratio is essential, although the effect of the operational conditions often promotes the proliferation of NOB [154]. In addition, high levels of heterotrophic, denitrifying bacteria inhabiting autotrophic nitrifying granules cultured without organic carbon sources supply have also been documented previously.

*Chloroflexi* and *Anaerolineaceae* used to be the most abundant denitrifying groups [150,155]. *Chloroflexi* facilitate complete denitrification using the expression of nitric oxide reductase and nitrite reductase genes and also promote biofilm aggregation in anammox granules, playing different roles depending on the operational conditions [23,39]. Both families have the ability to degrade organic matter, although it is suspected that *Anaerolineaceae* may be the first to degrade it [156]. Other phylotypes with lower abundance identified in anammox granules were *Acidobacteria*, *Actinobacteria*, *Bacteroidetes*, *Cyanobacteria*, *Firmicutes*, *Gemmatimonadetes*, *Spirochaetes*, and *Verrucomicrobia* [126,147]; however, genera such as *Thauera* can compete for available electron donors, interfering in the growth of complete denitrifier species [117]. The enrichment of *Thauera* enhances the priority of AnAOB microorganisms in competition for nitrite, improving the partial denitrification-anaerobic ammonia oxidation. The success of this genus can occur with the reduction of organic matter and shorter HRT [157]; therefore, the consortia *Nitrosospira* (AOB), *Thauera*, and Ca. Brocadia can be favored by short HRT [117].

## 5. Conclusions

Identification of the microorganisms involved in granular technologies for wastewater treatment is a necessary requirement to improve bioreactor operations and to take advantage of system functioning. Much correlational research has enhanced our understanding of the complex dynamics of the microbiota in granular biofilm, but functional studies are still lagging. Microbial activities are not isolated, but they are related to the presence and activity of surrounding microorganisms. These interactions between different kinds of microorganisms should be studied as functional entities to enhance pollutant removal and reduce exploitation costs. Each technology based on granular biofilms has a completely different microbiome; therefore, the importance of bioengineering tools lies in a deep knowledge of the microbiota, taking into account the effect of changes in operational conditions such as the concentration of ammonia, COD, micronutrients, and toxic pollutants, as well as conditions of feeding to the system. This review describes key microbial populations for pollutant degradation in granular technologies. A novel perspective on the functionality of microbial communities should be the focus of future research. In this sense, the future perspectives related to biological wastewater treatment systems should not only focus on the populations of microorganisms that inhabit the system through massive parallel sequencing studies but also on their activities using tools of molecular biology such as metatranscriptomic. Furthermore, currently, the study of microbial ecology in biological wastewater treatment is focused exclusively on prokaryotes, while eukaryotic microorganisms and viruses play a fundamental role in the stability of these ecosystems. Therefore, more effort must be made to have a complete framework of the microorganisms that inhabit these systems.
